# CXCL9 Contributes to Antimicrobial Protection of the Gut during *Citrobacter rodentium* Infection Independent of Chemokine-Receptor Signaling

**DOI:** 10.1371/journal.ppat.1004648

**Published:** 2015-02-02

**Authors:** Sarah A. Reid-Yu, Brian R. Tuinema, Cherrie N. Small, Lydia Xing, Brian K. Coombes

**Affiliations:** 1 Michael G. DeGroote Institute for Infectious Disease Research, Hamilton, Ontario, Canada; 2 Department of Biochemistry and Biomedical Sciences, McMaster University, Hamilton, Ontario, Canada; 3 Farncombe Family Digestive Health Research Institute, Hamilton, Ontario, Canada; The University of British Columbia, CANADA

## Abstract

Chemokines have been shown to be effective bactericidal molecules against a variety of bacteria and fungi *in vitro*. These direct antimicrobial effects are independent of their chemotactic activities involving immunological receptors. However, the direct biological role that these proteins may play in host defense, particularly against intestinal pathogens, is poorly understood. Here, we show that CXCL9, an ELR- chemokine, exhibits direct antimicrobial activity against *Citrobacter rodentium*, an attaching/effacing pathogen that infects the gut mucosa. Inhibition of this antimicrobial activity *in vivo* using anti-CXCL9 antibodies increases host susceptibility to *C. rodentium* infection with pronounced bacterial penetration into crypts, increased bacterial load, and worsened tissue pathology. Using Rag1^-/-^ mice and CXCR3^-/-^ mice, we demonstrate that the role for CXCL9 in protecting the gut mucosa is independent of an adaptive response or its immunological receptor, CXCR3. Finally, we provide evidence that phagocytes function in tandem with NK cells for robust CXCL9 responses to *C. rodentium*. These findings identify a novel role for the immune cell-derived CXCL9 chemokine in directing a protective antimicrobial response in the intestinal mucosa.

## Author Summary

Host defense peptides are an essential part of the innate immune response to pathogens, particularly at mucosal surfaces. Some chemokines, previously known for their ability to recruit immune cells to a site of inflammation, have been identified to have direct antimicrobial activity *in vitro* against a variety of pathogens. Despite this, it was unknown whether chemokines play a role in protecting the gut mucosa against enteric pathogens, independent of their immunological receptors. Using a mouse model of enteric pathogen infection with both wild type mice and genetic knockouts, we showed that the chemokine CXCL9 has direct antimicrobial activity against pathogen infection. This antimicrobial activity prevented the invasion of bacteria into intestinal crypts, thus protecting the host from immunopathology. Neutralization of this CXCL9-dependent antimicrobial activity increased host susceptibility to infection, leading to bacterial penetration into intestinal crypts and increased tissue pathology. These data support the importance of a receptor-independent role for chemokines in host defense at mucosal surfaces and may offer alternative treatment strategies for infections, particularly in regards to organisms that are resistant to conventional antibiotics.

## Introduction

The intestinal tract is a site of continuous interaction between host and microbe. Tight regulation of immune surveillance and activation maintains the integrity of this interface during non-infectious periods while preserving the ability to launch immediate action upon pathogen exposure. Chemokines are a vital component of this protective response, linking innate and adaptive immunity by activating and recruiting immune cells to infection sites [1]. Until recently, the function of these molecules was focused on their chemotactic activity induced upon interaction with their receptors on various immune cells [1]. However, mounting evidence has shown a direct antimicrobial function for a number of chemokines that relates to their cationic surface properties, similar to antimicrobial host defense peptides [2,3].

Host defense peptides (or antimicrobial peptides), are produced by a wide variety of cell types and form an important component of innate host defenses [4,5]. Although the exact bactericidal mechanism for cationic antimicrobial peptides remains debated [6], membrane-disrupting activity appears to be a common feature, facilitated by cationic charge distribution and amphipathicity allowing for attachment to and insertion into bacterial membranes. In mammals, antimicrobial peptides help protect the gut mucosae from infection and maintain intestinal homeostasis [7]. *In vitro*, the chemokine CXCL9 has potent bactericidal activity against *Escherichia coli*, *Listeria monocytogenes*, and *Bacillus anthracis* [3,8], likely related to a cationic C-terminal domain resembling well characterized antimicrobial peptides [3,9]. Additionally, antimicrobial activity of CXCL9 appears to play a key role in protection of mucosal surfaces against pathogen infection [8–11]. However, outside of its role in T cell homing and activation, a potential role for CXCL9 in conferring antimicrobial protection of the gut mucosae against intestinal infections has not been investigated.

*Citrobacter rodentium* is an intestinal pathogen of mice used to model infections with the attaching and effacing (A/E) human pathogens, enteropathogenic *E*. *coli* and enterohemorrhagic *E*. *coli* [12]. *C*. *rodentium* colonizes the cecum prior to traversing to the primary site of infection in the distal colon. Like other A/E pathogens, *Citrobacter* forms intimate attachments with the epithelial surface in the cecum and colon by using a type III secretion system and effectors that it injects into the host’s intestinal epithelial cells [13,14]. The host response to infection includes an early and robust chemokine response [15–17]. Infection clearance in resistant mice requires Th1 adaptive responses [13,18,19] mediated by the IFNγ-stimulated chemokines CXCL9, CXCL10, and CXCL11 acting in a CXCR3 dependent manner [20]. Interestingly, of all the ELR- chemokines, CXCL9 is the most highly expressed during *C*. *rodentium* infection [15], suggesting that its role in protecting the host from this intestinal pathogen might be dominant. Previous work by our laboratory found that depletion of IFNγ-producing NK1.1+ CD3- cells reduced CXCL9 levels in the *C*. *rodentium*-infected gut, leading to increased host susceptibility to infection [19]. Whether this was solely related to T cell recruitment or additional antimicrobial activity was not known. Here, we uncover a direct antibacterial function for CXCL9 that protects intestinal crypts from bacterial infiltration.

## Results

### CXCL9 directs *C*. *rodentium* killing *in vitro*

**Fig 1:**
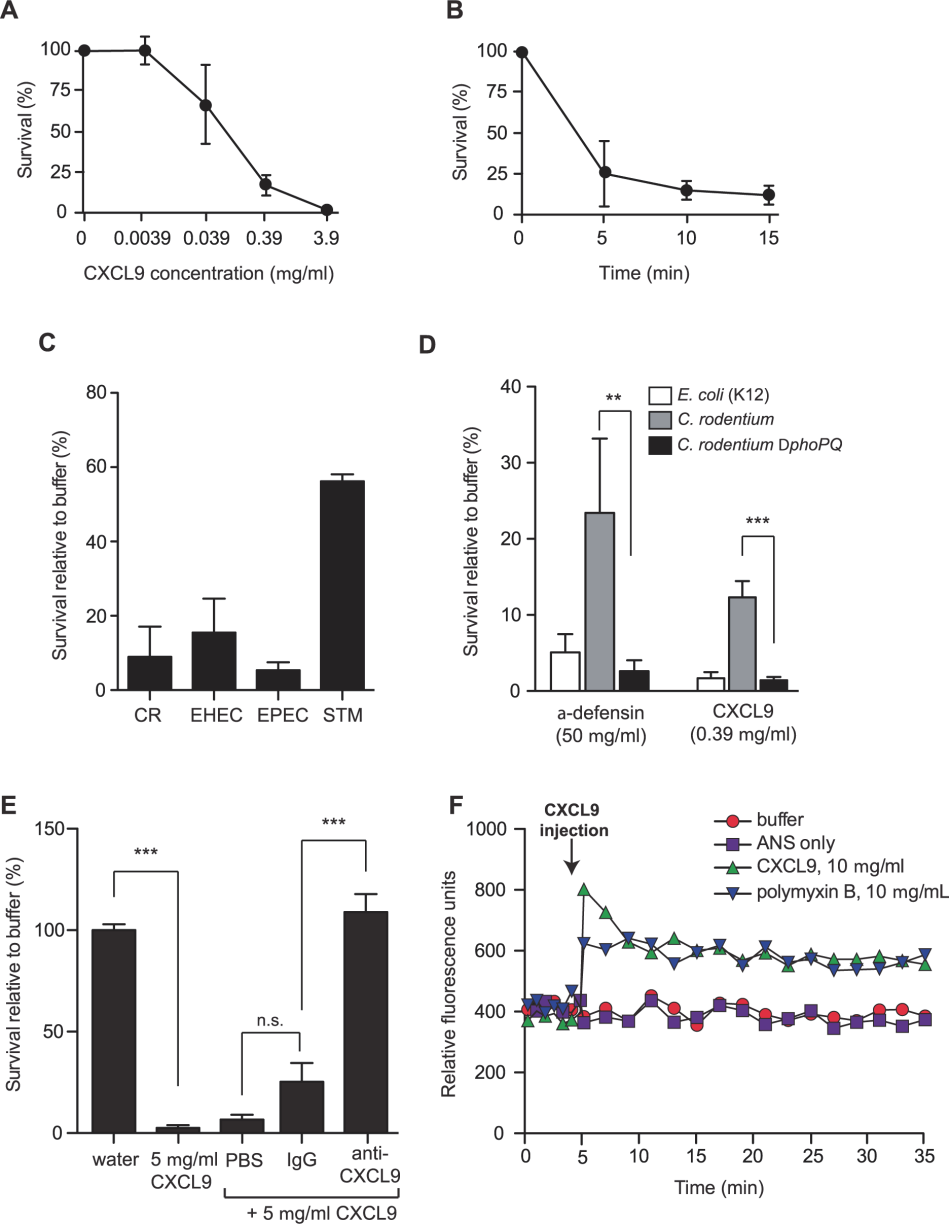
*Citrobacter rodentium* is sensitive to CXCL9-directed bacterial killing. **(A)** Dose response survival to increasing concentrations of CXCL9. Wild type *C*. *rodentium* was exposed to the various concentrations of CXCL9 for 2 h and survival was expressed as a percentage compared to buffer-only controls. Data are the means with standard error from 3 experiments. **(B)** *C*. *rodentium* time kill curves were plotted in response to 0.39 μg/ml CXCL9. Data are plotted as a percent survival compared to buffer controls without chemokine addition. Data are the means with standard error from 3 experiments. **(C)** Bacterial killing assays were performed in tandem with *C*. *rodentium* (CR), enterohemorrhagic *E*. *coli* (EHEC), enteropathogenic *E*. *coli* (EPEC), and *Salmonella enterica* serovar Typhimurium (STM) in the presence of 0.39 μg/ml CXCL9. Data is plotted as a percent survival compared to buffer controls. **(D)** PhoP-PhoQ is required for partial resistance to CXCL9-directed killing. The indicated strains were incubated with CXCL9 or α-defensin and survival was determined by viable colony counting. Data are the means with standard error from 3 experiments. **(E)** Anti-CXCL9 antibody blocks the killing activity of CXCL9. *C*. *rodentium* was incubated with CXCL9 in the presence of either PBS, IgG control, or anti-CXCL9 antibody. CXCL9-specific antibody blocked 100% of the killing activity. Data are the means with standard error from 3 experiments. **(F)** Membrane permeability in response to CXCL9 or the membrane-disrupting antimicrobial peptide polymyxin B was measured by ANS release. Arrow indicates time of CXCL9 or antimicrobial peptide injection. Data is representative of 3 independent experiments. Statistical significance was assessed utilizing an ANOVA with Newman-Keuls post-test.

ELR- chemokines have an emerging role as potent antimicrobial agents, owing to their cationic C-terminal domain rich in positively charged amino acids. Since the level of CXCL9 increases dramatically following *Citrobacter* infection of resistant mice [15], we first determined whether CXCL9 exerted antimicrobial activity against *C*. *rodentium in vitro*. Treatment of *C*. *rodentium* with CXCL9 resulted in a dose-dependent bacterial killing, as measured by viable colony counts of residual survivors (**Fig. 1A**). Exposure to CXCL9 at ∼4 μg/ml (270 nM) resulted in 100% killing, and 85% killing at ∼0.4 μg/ml (27 nM). This concentration is biologically relevant as CXCL9 levels in rectal perfusions from the inflamed human intestine can reach up to 2 μg/ml (138 nM) [21]. Time-kill curves showed that killing was rapid, reaching near maximal effect at ∼5 min post-exposure to 27 nM CXCL9 (**Fig. 1B**). Importantly, in addition to antimicrobial action against the mouse pathogen *C*. *rodentium*, we also observed similar susceptibility of human pathogens, EHEC and EPEC to CXCL9-directed bacterial killing (**Fig. 1C**).

Many host-adapted pathogens have evolved resistance to antimicrobial peptides through LPS modifications controlled by the two-component system PhoP-PhoQ [22–24]. To determine whether residual survival of *C*. *rodentium* following treatment with lower doses of CXCL9 was PhoP-PhoQ dependent, we treated wild type bacteria and a *phoPQ* deletion mutant with a concentration of CXCL9 that produced ∼10–20% survival of wild type bacteria, and measured viable bacteria after 1–2 h. The Δ*phoPQ* strain was more susceptible to CXCL9, with only 1–2% residual survivors, similar to a peptide-sensitive strain of *E*. *coli*. A similar result was seen using the human α-defensin, HD5, an abundant defense molecule produced by Paneth cells in the gut (**Fig. 1D**). Interestingly, we found that a 1000-fold lower molar concentration of CXCL9 could elicit similar killing when compared with HD5. To confirm that this killing was directly linked to CXCL9, we performed killing assays in the presence of purified anti-CXCL9 antibody or control IgG. Whereas CXCL9 killed ∼100% of *C*. *rodentium*, this activity was completely blocked by anti-CXCL9 antibody but not an IgG control (**Fig. 1E**). Antimicrobial peptides like polymyxin B kill bacteria by inducing membrane permeability [25], which can be measured by a fluorescence increase following membrane incorporation of the neutral hydrophobic molecule 1-anilino-8-naphthalene-sulfonate (ANS) [26]. Indeed, injection of polymyxin B into a culture of *C*. *rodentium* led to an immediate increase in ANS fluorescence (**Fig. 1F**). To better understand how CXCL9 might exert its antimicrobial activity, we measured ANS fluorescence following CXCL9 injection into *C*. *rodentium* culture. An increase in fluorescence, which occurs when ANS partitions into exposed membrane, was observed on a similar time scale and magnitude upon injection of CXCL9 as that seen with polymyxin B (**Fig. 1F**). Together, these data established a direct antimicrobial activity for CXCL9 on *C*. *rodentium* in a manner consistent with membrane disruption, similar to classic antimicrobial peptides.

### CXCL9 increases host survival and decreases *C*. *rodentium* burden *in vivo*

**Fig 2:**
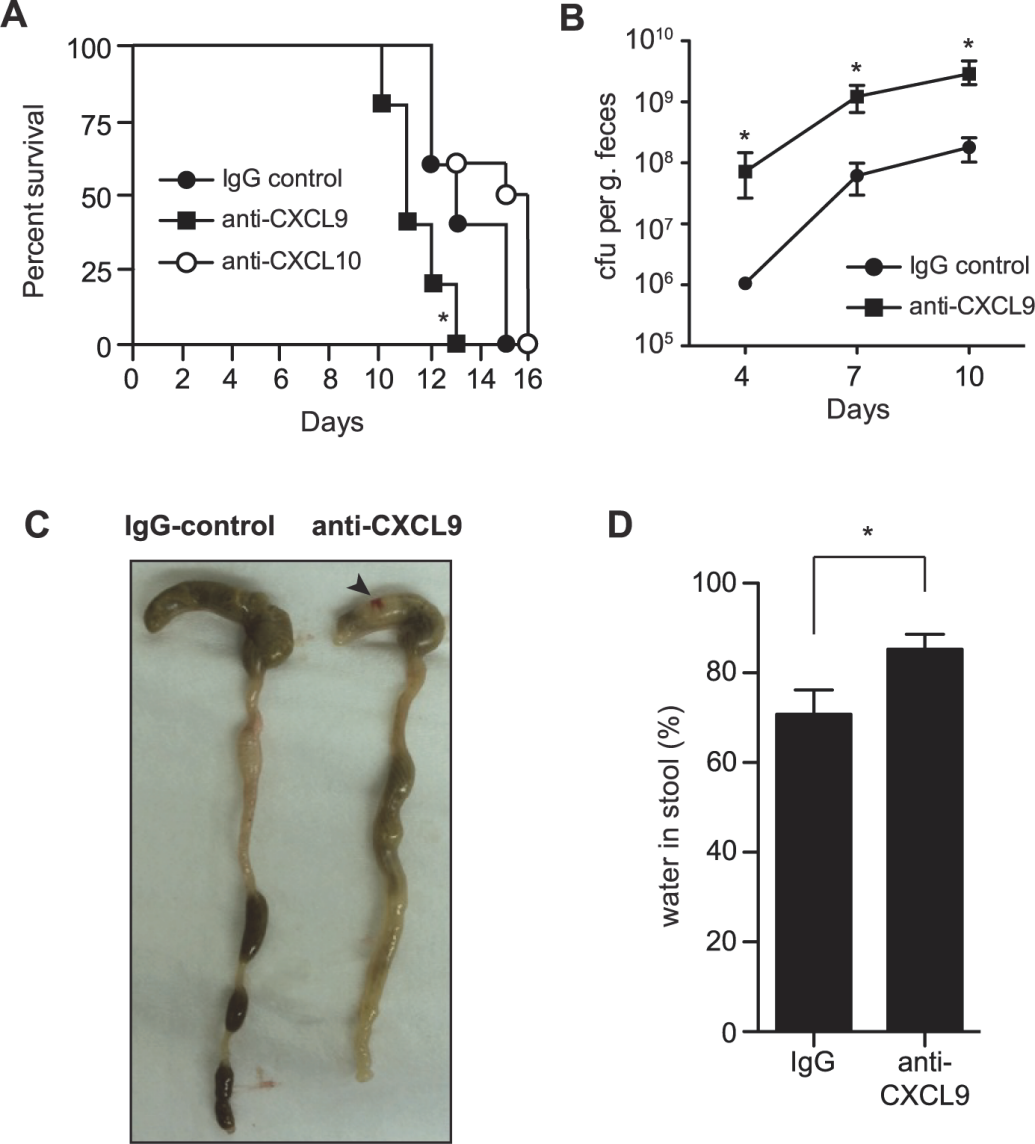
Loss of CXCL9 results in increased *C*. *rodentium* burden and worsened host outcome. (**A**) Rag1^-/-^ mice were infected with *C*. *rodentium* and administered control rabbit IgG, anti-CXCL9 antibody, or anti-CXCL10 antibody. Survival was monitored for 15 days. Data are from 2 independent experiments. * *P* <0.05 (Gehan-Breslow-Wilcox) compared to IgG control. **(B)** Rag1^-/-^ mice were infected as above and viable *C*. *rodentium* counts were determined in fecal pellets. Data are the means with standard errors from 5 animals. * *P* <0.05 compared to IgG control. **(C)** Representative images of gross pathology in the cecum and colon of Rag1^-/-^ mice infected with *C*. *rodentium* for 10 days. Note increased water content in mice depleted of CXCL9, emptied and shrunken cecum and constricted colon. Arrowhead indicates hematoma, which was common in infected CXCL9-depleted animals. **(D)** Water content in fecal output was determined at day 10 after infection. Data is pooled from three separate experiments (9 animals) * *P*<0.05. Statistical significance was assessed utilizing the t-test.

Previous work investigating the role of CXCL9 in the gut has focused solely upon its chemotactic properties. However our *in vitro* results suggested that CXCL9 might have biological significance as a direct antimicrobial molecule. To investigate this, we first measured the levels of CXCL9 in the gut following *C*. *rodentium* infection by ELISA. CXCL9 was readily detectable in a variety of tissue samples, including fecal pellets, rectal perfusions, scrapings of the colonic mucosae, and in total homogenized colon samples (**S1A Fig.**). Next, we depleted CXCL9 in Rag1^-/-^ mice infected with *C*. *rodentium* and monitored host survival and bacterial load. Rag1^-/-^ mice were used in order to study the protective effect of CXCL9 independent of its ability to recruit T cells. *C*. *rodentium*-infected mice depleted of CXCL9 died, on average, 2 days earlier than mice receiving IgG control. This was significant, and specific to CXCL9 depletion, as depletion of another antimicrobial ELR- chemokine, CXCL10, had no effect on host mortality over 10 days (**Fig. 2A**). This difference between CXCL9 and CXCL10 in affecting disease severity may be due to the differential expression of the two chemokines, where CXCL9 is expressed at much higher levels in the infected colon, suggesting a more dominant role [15]. In line with these data, *C*. *rodentium* burden was 10–100 times higher in the fecal output from CXCL9-depleted mice compared to controls over the 10-day infection period (**Fig. 2B**). In order to confirm that anti-CXCL9 antibodies were reaching the lumen of the gut, we measured IgG levels in fecal samples following intraperitoneal delivery of anti-CXCL9 antibody or control IgG into uninfected Rag1^-/-^ mice and showed an IgG accumulation in the feces (**S1B Fig.**). As a secondary biological readout for CXCL9 depletion, we measured the number of infiltrating CD3+ cells in the distal colon 10 days after *C*. *rodentium* infection of immunocompetent C57BL/6 mice and in mice depleted of CXCL9. As expected, in *C*. *rodentium*-infected immunocompetent C57BL/6 mice depleted of CXCL9, there was a ∼75% reduction in the number of CD3+ cells in the distal colon (**S1C–D Fig.**).

To investigate the impact of CXCL9 depletion on disease severity, we examined the gross pathology of the gut during necropsy. *C*. *rodentium*-infected mice depleted of CXCL9 had (*i*) increased evidence of colitis in the cecum, which was shrunken and partially emptied; (*ii*) shortening and thickening of the colon; (*iii*) increased incidence of watery stool; and (*iv*) hematomas along the length of the cecum and colon (**Fig. 2C**). As a measure of diarrhea in the stool, we measured fecal water content at day 10, which was significantly greater in CXCL9-depleted mice compared to controls (**Fig. 2D**). These results were independent of significant differences in the levels of TNFα, IFNγ, IL12p40, or IL-10 in colonic explants on day 10 post-infection, which were all similar in control mice and mice depleted of CXCL9 (**S2 Fig.**). Together, these data established an important role for CXCL9 in host defense against *C*. *rodentium* infection and in limiting *C*. *rodentium* burden in the gut.

### CXCL9 protects the gut from *C*. *rodentium*-induced pathology and bacterial penetration into crypts

**Fig 3:**
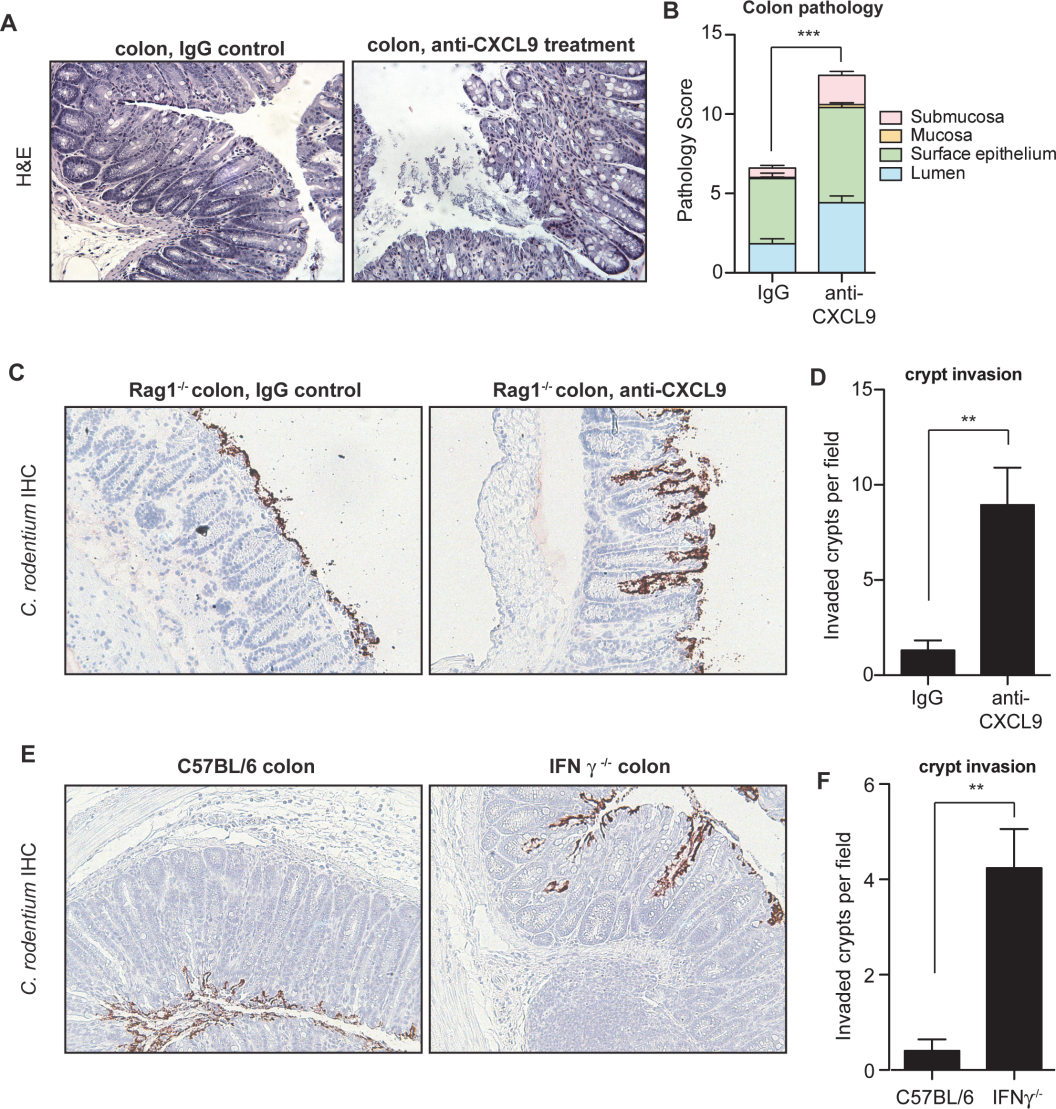
Mice depleted of CXCL9 have greater *C*. rodentium-induced pathology. **(A)** Rag1^-/-^ mice were infected with *C*. *rodentium* and administered IgG control antibody or anti-CXCL9 antibody. Representative histopathology images (200x) of the distal colon are shown from animals infected for 10 days. **(B)** Quantification of pathology in colon. Data is pooled from two experiments, n = 6 per group. **(C)** Localization of *C*. *rodentium* in Rag1^-/-^ mouse colon by immunohistochemistry. Mice were infected with *C*. *rodentium* for 10 days. Images (200x) are representative of two separate experiments, n = 6 per group. (**D**) Quantification of crypt invasion by *C*. *rodentium* in Rag1^-/-^ mice. Data are the means with standard errors. **(E)** Localization of *C*. *rodentium* in the distal colon in C57BL/6 mice and IFN-γ^-/-^ mice by immunohistochemistry. Mice were assessed on day 10 after *C*. *rodentium* infection. Images (200x) are representative of two separate experiments, n = 5 per group. (**F**) Quantification of crypt invasion by *C*. *rodentium* in IFNγ^-/-^ mice. Data are the means with standard errors. Statistical significance was assessed utilizing the t-test. **P<0.01; ***P<0.001.

The gross pathological differences in *C*. *rodentium*-infected mice upon CXCL9 depletion suggested a worsened immunopathological response to infection, which correlated with increased bacterial load. Since the pathologic impact on the host can be affected by less than a log-change in peak *C*. *rodentium* load [27], we scored colonic histopathology on day 10 in *C*. *rodentium*-infected control mice and animals depleted of CXCL9. CXCL9 depletion was associated with increased numbers of sloughed epithelial cells in the lumen and immune cell infiltration, with destruction of the epithelial architecture (**Fig. 3A**). These changes resulted in significantly greater transmural pathology in the colon (**Fig. 3B**). Similar findings were observed for cecal pathology upon *C*. *rodentium* infection of CXCL9-depleted mice, with a more pronounced pathology in the mucosa and submucosa regions of depleted animals (**S3 Fig.**). In a typical *C*. *rodentium* infection, the bacteria attach to the intestinal epithelium, but do not commonly penetrate deep into intestinal crypts [28,29]. To localize *C*. *rodentium* in infected mice in the presence or absence of CXCL9, we performed immunohistochemical localization of *C*. *rodentium* using an antibody specific to *C*. *rodentium* LPS. Indeed, we observed the majority of *C*. *rodentium* in close association with the colonic epithelial cell surface in uncontrived Rag1^-/-^ mice, with only marginal evidence of crypt penetration. In contrast, in mice depleted of CXCL9, *C*. *rodentium* was commonly found to penetrate deeply into crypts in the colon (**Fig. 3C and 3D**) and cecum (**S3 Fig.**).

CXCL9 is mainly dependent on IFNγ for its expression [30], and IFNγ^-/-^ mice have impaired resistance and greater pathology following *C*. *rodentium* infection similar to that seen in our CXCL9 depletion studies [31]. Given our results following infection of CXCL9-depleted mice, we hypothesized that IFNγ^-/-^ mice would be similarly susceptible to crypt penetration by *C*. *rodentium* due to the attendant decrease in CXCL9 expression. We tested this by localizing *C*. *rodentium* in colonic tissues of IFNγ^-/-^ mice and C57BL/6 wild type controls by immunohistochemical staining. In these experiments, we found that *C*. *rodentium* was localized mainly to the epithelial surface in C57BL/6 mice, whereas bacteria were commonly found penetrating into colonic crypts of IFNγ^-/-^ mice (**Fig. 3E and 3F**). Together, these data indicated that the antimicrobial action of CXCL9 helps maintain epithelial barrier defenses against *C*. *rodentium* by preventing crypt penetration by invading bacteria. The loss of this defense upon CXCL9 depletion allows for bacterial penetration deep into intestinal crypts with an attendant increase in pathology.

### The antibacterial defense of CXCL9 is independent of the CXCR3 chemokine receptor

**Fig 4:**
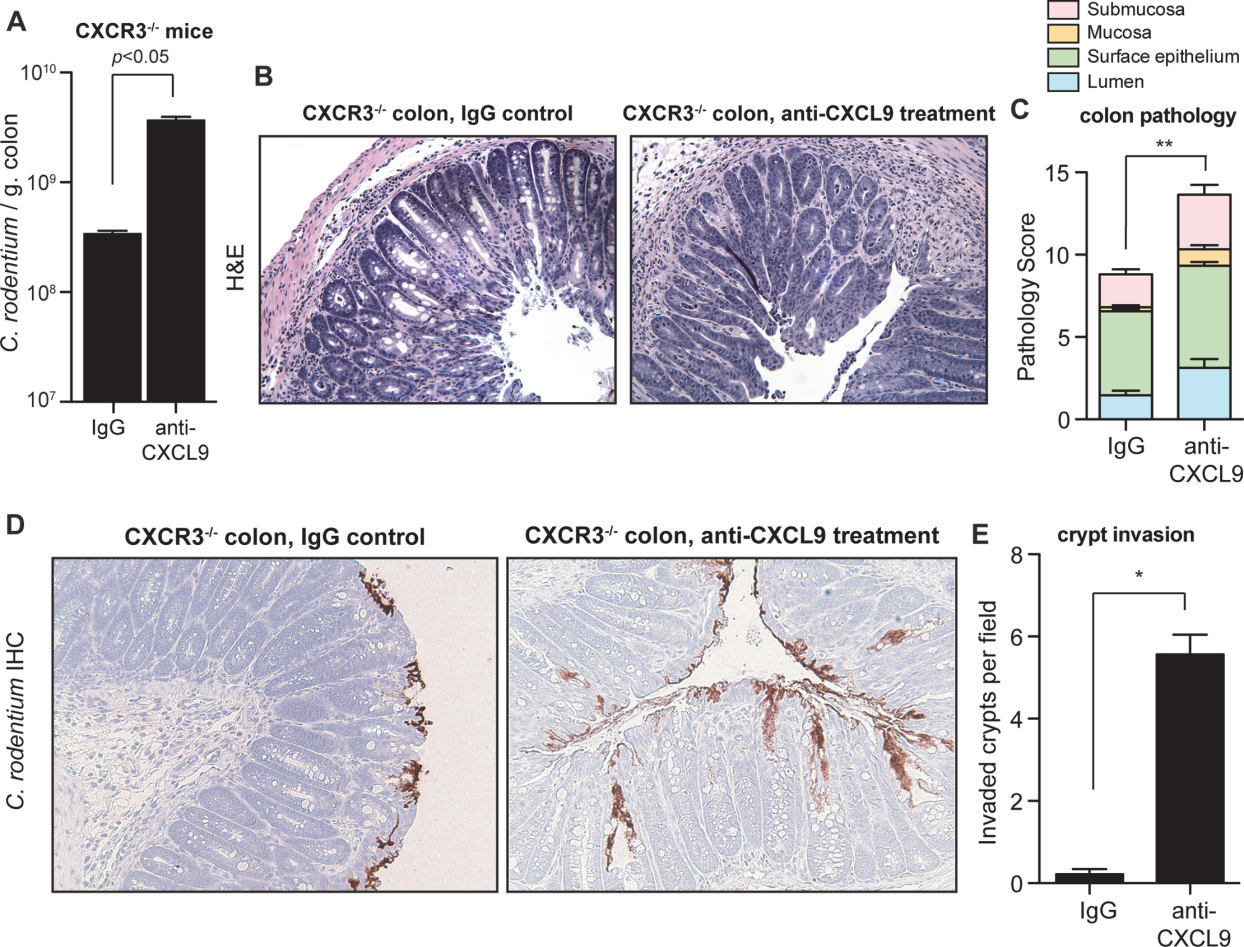
CXCL9-mediated host protection against *C*. *rodentium* is independent of CXCR3. **(A)** CXCR3^-/-^ mice were infected with *C*. *rodentium* and tissue-associated bacterial burden was assessed on day 10 after anti-CXCL9 treatment, or control IgG treatment. Data are the means with standard error from 3 experiments. **(B)** Representative H&E-stained colon sections (200x) from *C*. *rodentium*-infected CXCR3^-/-^ mice. Images are representative from 2 experiments, n = 4 per group. **(C)** Pathology scores in the distal colon of CXCR3^-/-^ mice infected with *C*. *rodentium*. **(D)** Localization of *C*. *rodentium* in uncontrived CXCR3^-/-^ mice (left panel) or mice depleted of CXCL9 (right panel). Immunohistochemistry images (200x) are representative of 2 experiments, n = 4 per group. **(E)** Quantification of crypt invasion by *C*. *rodentium* in CXCR3^-/-^ mice. Data are the means with standard errors. Statistical significance was assessed utilizing the t-test. *P<0.05; **P<0.01.

To firmly establish the biological significance of direct antimicrobial activity of CXCL9 during *C*. *rodentium* infection, we infected CXCR3^-/-^ mice that lack the CXCL9 chemokine receptor and thus do not mount CXCR3-dependent responses following ligand interactions. Based on our prior data, we hypothesized that the host susceptibility to *C*. *rodentium* infection following CXCL9 depletion would persist in CXCR3^-/-^ mice. Indeed, CXCR3^-/-^ mice depleted of CXCL9 carried a significantly increased burden of tissue-associated *C*. *rodentium* in the colon (**Fig. 4A**). Furthermore, CXCL9-depleted CXCR3^-/-^ mice had increased pathology scores in both the distal colon (**Fig. 4B** and **Fig. 4C**) and in the cecum (**S4 Fig.**). In agreement with our previous data for a direct role for CXCL9-mediated protection of intestinal crypts, uncontrived CXCR3^-/-^ mice were able to restrict *C*. *rodentium* to the epithelial cell surface with virtually no penetration by bacteria into intestinal crypts. In contrast, depletion of CXCL9 in *C*. *rodentium*-infected CXCR3^-/-^ mice produced a striking invasion of bacteria deep into intestinal crypts in both the colon (**Fig. 4D and 4E**) and the cecum (**S4 Fig.**). These data confirmed that the antibacterial activity and host protection afforded by CXCL9 was independent of CXCR3-ligand-mediated effects.

### IFNγ produced by NK cells and macrophages is necessary to achieve maximal CXCL9 expression in response to *C*. *rodentium*

**Fig 5:**
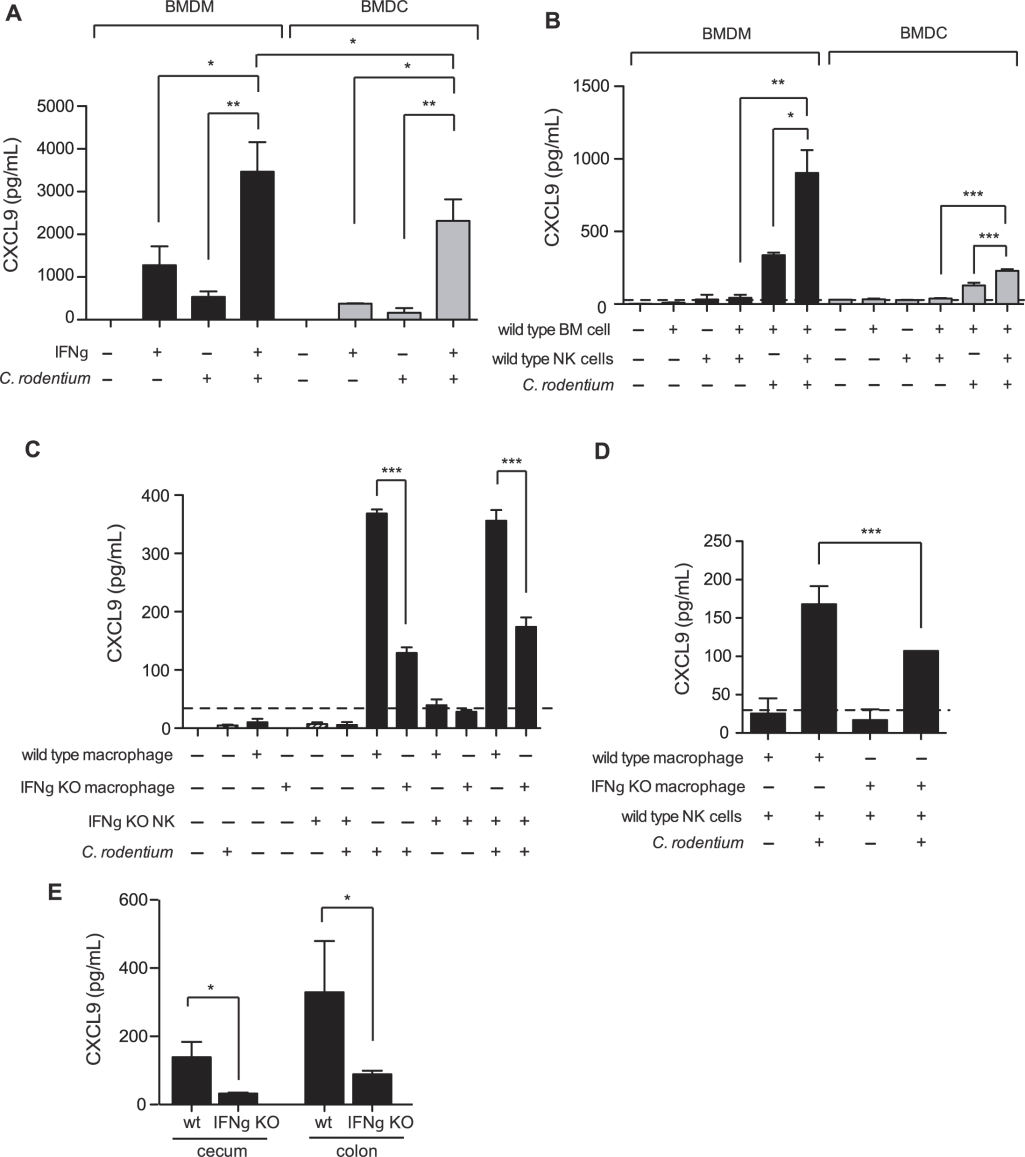
Macrophages require NK cells and IFNγ for optimal CXCL9 production. **(A)**. Bone marrow-derived macrophages (BMDM), or dendritic cells (BMDC) were incubated for 24 h at 37°C with either 1 ng/mL IFNγ, and/or heat killed (hk) *C*. *rodentium*. CXCL9 release was determined from cell culture supernatants by ELISA. Data is the mean with standard error from three experiments. **(B)**. Co-culture of BMDM or BMDC with NK cells leads to increased release of CXCL9. Data is the mean with standard error from three experiments. (**C and D**). 5x10^4^ BMDM, or BMDC from C57BL/6 (WT) or IFNγ^-/-^ were incubated for 24 h at 37°C with C57BL/6 (WT) or IFNγ^-/-^ NK cells, hk *C*. *rodentium*, or media alone, as indicated. Data is the mean with standard error from three experiments. (**E)**. CXCL9 expression was determined from cecal or colonic explant supernatants from *Citrobacter*-infected C57BL/6 (WT), or IFNγ^-/-^ mice, day 10 p.i. Explant data is from 5 separate animals per group. Significance was determined using an ANOVA with a Newman Keuls post test (A-C), or a Student’s t test (D-E). *P<0.05; **P<0.01; ***P<0.001.

Previous work examining transcript levels of CXCL9 in the *C*. *rodentium* infected colon found the predominant source to be CD11c+ cells, and was therefore attributed dendritic cells (DCs) [15]. However, recent work into understanding the role that DCs and macrophages play in intestinal homeostasis, as well as inflammation, has revealed that the intestinal tract is more heavily populated with macrophages [32], and that some previous work attributing function to CD11c+ DCs has instead been misidentified macrophages [33–35]. Indeed, macrophages have been shown to be a significant source of CXCL9 in other inflamed tissues [36,37]. We measured CXCL9 release from GM-CSF induced bone marrow-derived DC (BMDC) and M-CSF induced macrophages (BMDM) in response to heat-killed *Citrobacter* and IFNγ. Unstimulated BMDC and BMDM did not produce detectable levels of secreted CXCL9. However, exposure to either IFNγ alone, or *C*. *rodentium* alone stimulated intermediate levels of CXCL9, which was significantly boosted in response to a combination of both stimuli (**Fig. 5A**). These data were consistent with that of others, showing that maximal CXCL9 expression is induced by IFNγ, in combination with additional microbial stimuli [38,39].

We examined the potential sources of IFNγ responsible for driving CXCL9 expression within DCs, and macrophages. It is well established that natural killer (NK) cells are an important early source of IFNγ, and previous work by our laboratory has shown that depletion of NK1.1+ cells, a common NK cell marker, in *Citrobacter*-infected mice results in decreased CXCL9 expression in the colon [19]. Unstimulated BMDM, BMDC, or NK cells did not express CXCL9. However, co-culture of BMDM and NK cells, or DCs and NK cells, lead to CXCL9 secretion that was dependent on the presence of *C*. *rodentium* (**Fig. 5B**). NK cells were important for maximal CXCL9 release as CXCL9 levels were reduced by ∼50% in the absence of NK cells. Interestingly, we observed that macrophages were far more capable at not only inducing their own expression of CXCL9 upon exposure to *C*. *rodentium*, independent of NK cells, but also released more CXCL9 upon co-stimulation with either IFNγ and *Citrobacter*, or NK cells and *Citrobacter* in paired experiments directly comparing macrophages and inflammatory DC populations (**Fig. 5B**).

Previous studies have shown that macrophages are capable of producing IFNγ in a TLR signaling-dependent fashion [40,41]. Using NK cells isolated from wild type mice, or IFNγ^-/-^ animals and BMDM derived from these mice, we measured the contribution IFNγ produced by macrophages and NK cells on CXCL9 release following *C*. *rodentium* stimulation. We found that IFNγ expression by both NK cells and macrophages was critical for maximum CXCL9 release in the presence of heat-killed *C*. *rodentium* (**Fig. 5C and 5D**). Equivalent CXCL9 levels were released from *C*. *rodentium*-stimulated BMDM from wild type mice when NK cells were absent, or in the presence of IFNγ-deficient NK cells. These data indicated that the release of CXCL9 was dependent upon IFNγ expression by NK cells, and not other co-stimulatory, and/or NK cell-directed alternative cytokine expression mechanisms. Macrophage-derived IFNγ was also important for this response as release of CXCL9 was significantly reduced by ∼50–60% from macrophages unable to produce their own IFNγ (**Fig. 5D**). This was further supported in experiments that showed CXCL9 release from *C*. *rodentium*-stimulated IFNγ^-/-^ macrophages was significantly less than CXCL9 levels from wild type macrophages (**Fig. 5C**). Together, these data indicated that IFNγ produced by both NK cells and macrophages is necessary to achieve maximal CXCL9 expression in response to *C*. *rodentium*. Finally, we measured CXCL9 levels in the gut following *C*. *rodentium* infection and found that, similar to our *in vitro* results, chemokine levels in the cecum and colon were significantly blunted in IFNγ^-/-^ mice (**Fig. 5E**). Together, these data indicate that IFNγ produced by NK cells and macrophages is necessary to achieve maximal CXCL9 expression in response to *C*. *rodentium*.

## Discussion

The intestinal tract is an environment that requires a balanced set of immunological responses, capable of tolerating the host’s commensal microbiota, while remaining primed to respond to invasion by pathogenic bacteria. Early immune responses to pathogens are critical for controlling both bacterial burden, and disease pathology, which the host achieves through combined cellular and innate antimicrobial responses. Chemokines are an important facet to this host protection, by linking innate antimicrobial activity with cell homing to the site of infection. We found that CXCL9, a chemokine known previously as an important modulator of CXCR3-dependent cellular homing following *C*. *rodentium* infections, has an important additional function in innate antimicrobial defense of the gut. This antimicrobial activity is independent of the CXCR3 receptor, or other aspects of adaptive immunity, and helps to control bacterial burden while protecting intestinal crypts from pathogen invasion.

IFNγ^-/-^ mice are more susceptible to *C*. *rodentium* infection [31]. The basis for this was thought to be the loss an IFNγ-dependent antimicrobial factor expressed in the colon. Our results are consistent with CXCL9, a chemokine induced by IFNγ in combination with other microbial stimuli, as a likely mediator of host protection in IFNγ-competent hosts. Previous worked showed that p38α expression in T cells regulates host defense against *C*. *rodentium* infection [42]. This study revealed that T cells lacking p38α had a significant reduction in IFNγ production following *C*. *rodentium* infection, a decreased infiltration of inflammatory cells into the colon, and yet increased tissue damage, a result that could be linked to the increased invasion of *C*. *rodentium* in mice with p38α T cell deficiency. Interestingly, treatment of these mice with IFNγ restored host defenses against *C*. *rodentium*, leading to lessened tissue damage, and more importantly, normalization of the tissue-associated bacterial burden. While the authors attributed these findings to increased T cell homing to site of infection, an alternative or additional interpretation, given our current results, is the attendant increase in innate defense mediated by IFNγ-stimulated CXCL9 release.

The increased tissue damage resulting from loss of IFNγ, despite the blunted immune cell infiltration [19,42], is likely due to the pathogen itself gaining access to the privileged host niche within intestinal crypts. In a typical *C*. *rodentium* infection of resistant hosts, the host restricts the pathogen to the lumen or epithelial surface. However, depletion of CXCL9, or loss of IFNγ production allows for *C*. *rodentium* to penetrate deep within the crypts. A correlation between invasion of *C*. *rodentium* into crypts and increased host pathology has been observed in other studies [43,44] and so protecting this niche against intestinal pathogens is a key function for the innate immune system.

Of note, we found that IFNγ^-/-^ mice were capable of producing a modest, but non-protective level of CXCL9 in the colon following *C*. *rodentium* infection. Although CXCL9 production is typically considered to be dependent on IFNγ, an alternative induction pathway has been described macrophages involving IFNα/ signaling. For example, low-level expression of CXCL9 was described in IFNγ^-/-^ mice following infection with vaccinia virus [45]. In addition, STAT1 activation by IFNα in primed macrophages also boosts CXCL9 expression [46]. The quantitative contribution of this IFNγ-independent production of CXCL9 on host protection, however, has not yet been defined.

In this work, we observed that macrophages exhibited greater capacity for CXCL9 expression in the presence of microbial stimuli, and/or IFNγ, compared to DCs. Previous observations attributed the greatest levels of CXCL9 transcript to CD11c+ DCs in the *Citrobacter*-infected colon [15]. However this study relied upon CD11b and CD11c markers to differentiate phagocytes, typical surface markers routinely used to identify DCs and macrophage populations. However, recently it has become clear that many markers previous attributed to a homogenous phagocyte populations, in particular CD11c and CD11b, are expressed on multiple cell types [33]. Therefore, further examination, and in particular direct cell staining for CXCL9 expression, is necessary to identify the population(s) essential for robust expression of this chemokine. In addition to DCs, various cell types have been found to express CXCL9, including epithelial cells, neutrophils, and macrophages [36–38,47]. Recently, staining for CXCL9-producing cells within the inflamed tonsils also revealed macrophages to be the predominate source of the chemokine [37]. Preliminary data from our laboratory has shown co-localization of CXCL9 expression with the F4/80 macrophage marker within the colon of *C*. *rodentium* infected mice (data not shown). Given these data, macrophages appear to be a significant source of CXCL9 within the inflamed colon of *C*. *rodentium* infected mice, however the utility of current molecular tools to investigate the cellular sources of CXCL9 in the gut appear, in our hands, to be limited. This could potentially be overcome by directly labeling native CXCL9 in transgenic mice [48], however additional work is required.

Some host-adapted bacteria have evolved mechanisms of resistance towards antimicrobial host defense peptides through enzymatic cleavage-based mechanisms [49,50]. Evidence for bacterial resistance to the antibacterial activity of CXCL9 has also been observed, further implicating it as an innate host defense that can be a selectable target of resistance. For instance, the streptococcal inhibitor of complement (SIC) protein, secreted by *Streptococcus pyogenes*, can inhibit the antimicrobial activity of the CXCL9 C-terminal domain [9]. SufA from *Finegoldia magna* can also block the antimicrobial activity of CXCL9 by cleavage, while leaving its chemotactic activity intact [10]. *C*. *rodentium* does not appear to have such intrinsic resistance mechanisms; however, it is possible that other host-derived mechanisms may play a role in certain infections. For example, interaction of *Streptococcus dysgalactiae* with human serum albumin blocks some CXCL9-directed killing activity [51]. Whether such a mechanism is relevant in intestinal infections is not known.

In summary, our data indicate that CXCL9 plays an important role in antimicrobial defense in the infected and inflamed gut. This activity, independent of the chemokine receptor CXCR3 or an adaptive immune response, protects the gut from crypt invasion by *C*. *rodentium* and the tissue damage that ensues. These data add to the growing body of evidence to support this chemokine as an innate antimicrobial defense molecule at mucosal surfaces.

## Materials and Methods

### Ethics statement

All animal experiments were conducted according to guidelines set by the Canadian Council on Animal Care using protocols approved by the Animal Review Ethics Board at McMaster University.

### Animal infections

Six to eight-week old C57BL/6, Rag1^-/-^, IFNγ^-/-^, and CXCR3^-/-^ mice were purchased from Jackson Laboratories. Survival experiments were performed with 4-week old Rag1^-/-^ mice. All animals were housed in a specific pathogen-free unit under Level 2 conditions at the Central Animal Facility at McMaster University. For all infections, mice received 2 x 10^8^ CFU/mL via orogastric gavage from an overnight culture of *Citrobacter rodentium* (DBS100). For infections, bacteria were pelleted, washed, and resuspended in 10 mM HEPES (pH 8.0), 0.9% NaCl. Bacterial burden was monitored at designated time points in feces throughout experiments. At day 10 post infection, mice were sacrificed, and *C*. *rodentium* burden was determined in the cecum and colon as previously described [19]. For antibody neutralization experiments, mice were given either 200 μg/mL rabbit anti-mouse CXCL9, or 200 μg/mL control rabbit IgG on day -1, 0, 1, and then every 3 days via intraperitoneal injection. All neutralization antibodies were column-purified from rabbit antisera, which was a kind gift from Dr. Cory Hogaboam (University of Michigan/Cedars-Sinai Medical Center).

### *In vitro* killing assay

Bacterial killing assays were performed with wild type *C*. *rodentium*, enterohemorrhagic *E*. *coli*, enteropathogenic *E*. *coli*, *S*. Typhimurium, a Δ*phoPQ C*. *rodentium* mutant, and *E*. *coli* K12. The *phoPQ* deletion was generated by Lambda Red mutagenesis according to published methods [52] using primers BRT151 (tta gcc gtc ctt ctg ccc cgg ctg ctg tcg gcc aaa aat gac ctc cat gtg tag gct gga gct gct tcg) and BRT152 (atg cgc gtt ctg gtt gtt gag gat aat gcg tta cta cgt cac cac ctg cat atg aat atc ctc ctt a). Stationary phase (16–18h) cultures were sub-cultured 1:50 in LB, and grown at 37°C with shaking until OD_600_ = 0.5. Bacteria were pelleted, washed and resuspended in 10 mM HEPES buffer (pH 7.4) to a concentration of 10^5^ CFU/mL. Killing was initiated by mixing bacteria with 50 μg/mL human α-defensin (HD5; Prospec), CXCL9 (0.39 μg/mL, or otherwise indicated concentration; Peprotech), or sterile water. Bacteria were incubated at room temperature for 2 h, unless otherwise indicated. Cultures were diluted 1:10 with PBS to quench killing, and viable bacterial counts were assessed on solid agar. All data was normalized to the water control and expressed as survival relative to time zero. For CXCL9 neutralization in the bacterial killing assays, 200 μg/mL antibody (or similarly diluted PBS) was pre-incubated with 5 μg/mL CXCL9 for 20 min prior to the assay.

### ANS membrane permeability assay

To assess integrity of bacterial cellular membranes, the fluorescent probe, 8-anilino-1-naphthylenesulfonic acid (ANS; Sigma-Aldrich) was used according to previous published protocols [53]. In brief, stationary phase cultures of *C*. *rodentium* were sub-cultured 1:50 in LB, and grown at 37°C with shaking until OD_600_ = 0.5. Bacteria were pelleted, washed, and resuspended in sterile 10 mM HEPES Buffer (pH 7.4), 5 μM carbonyl cyanide 3-chlorophenylhydrazone (CCCP; Sigma Aldrich), and 5 mM glucose. Bacteria were incubated for 30 min at room temperature. For each sample, 93 μL of bacteria was added to each well of black, clear bottom, 96-well plate (Costar, Corning, Inc.) with 2 μL 3 mM ANS, and fluorescence was monitored on a Synergy HT microplate reader (BioTek) (excitation, 375nm; emission, 510nm). After 5 min, 10 μg/mL CXCL9, 10 μg/mL Polymyxin B (Sigma-Aldrich), or water control was injected and fluorescence was monitored for an additional 30 min.

### Histochemical analysis

At 10 days post infection, segments of cecal tip or distal colon were collected and either fixed in buffered 10% formalin, or flash frozen in optimal cutting template compound (OCT; Sakura, Fisher). Segments were fixed for 72 h, paraffin-embedded, sectioned into 6 μm slices, and stained with hematoxylin and eosin (H&E), anti-CD3 antibody (1:1000; Labvision), or anti-*Citrobacter* antibody (1:4000; Statens Serum Institute). H&E sections were used for assessing pathology according to published scoring protocols [54]. All fixed sections were visualized using a Leica microscope. A minimum of 6 views were analyzed for each sample. Evaluation of *C*. *rodentium* crypt invasion was determined through enumeration of bacterially penetrated crypts from 4–6 views per sample. Anti-CD3 treated sections were enumerated using ImageJ software.

### CXCL9 quantification

At day 8 post infection, wild type C57BL/6 mice were euthanized, and CXCL9 levels were detected in various tissues. Rectal perfusions were performed with 0.5mL PBS with protease inhibitors (PBS-I, 10 mL PBS per 1 tablet complete Mini, EDTA free (Roche)). Colon was excised, flushed with 5 mL ice cold PBS, and then opened longitudinally. Mucus scrapings were performed by running a razor blade along the length of the open colon according to established protocols [55]. Collected scrapings were diluted 1:10 in PBS-I, and vortexed for 3 min. Remaining colonic tissue was placed in 0.5 mL PBS-I with a metal ball and homogenized in a Mixer Mill (Retsch). Fecal pellets were similarly homogenized in 0.5 mL PBS-I. Solid particulates from all homogenized samples were pelleted through centrifugation prior to addition to ELISA plate wells. CXCL9 was quantified using a Duoset murine CXCL9 ELISA (R&D Systems) according to the manufacturer’s protocol.

### Cytokine and IgG quantification

At day 10 post infection, cecum and colon were removed, flushed of contents, and washed in ice-cold PBS, pH 7.4. Tissues were cut into 5 mm pieces, and placed in 1 mL RPMI, 50 μg/mL gentamicin. Tissues were incubated for 24 h at 37°C, 5% CO_2_. Levels of CXCL9, IFNγ, TNFα, IL-12p40, and IL-10 were assessed using Duoset Quantikine murine ELISA kits (R&D Systems) according to the manufacturer’s protocols. For IgG analysis of fecal pellet homogenate supernatants, fresh fecal pellets were collected on day 2 post final injection from naïve, uninfected animals receiving two injections of 200 μg/mL anti-CXCL9 antibody, control IgG, or PBS. Fecal pellets were homogenized as described above, and then centrifuged at 14,000 *g* for 20 min. Supernatants were collected, and frozen at -80°C until analysis. IgG ELISA was performed on samples by coating high-binding 96-well plates overnight at room temperature with goat anti-rabbit anti-IgG antibody (1:10,000; MP Bio). Plates were washed 3 times with PBS (pH 7.4), 0.05% Tween-20, and then blocked with PBS, 1% BSA for 1 h at room temperature. Plates were washed, and samples were added and incubated for 2 h at room temperature. For each sample, a 1:2 dilution (in PBS, 1% BSA) was made, and 100 μL was added to each well. After incubation, the plate was again washed three times. Bound antibody was detected by addition of anti-rabbit IgG antibody conjugated to HRP (1:10,000; GE Healthcare), and incubated at room temperature for 2 h. Plates were washed, and developed with 100 μL solution A and B (R&D Systems) for 20 min. The reaction was stopped by addition of 50 μL 1N H_2_SO_4_. Plates were read at 450 nm using a plate reader.

### *In vitro* co-culture of bone marrow-derived cells and NK cells

NK cells were purified from whole splenocytes from wild type C57BL/6 or IFNγ^-/-^ mice using a NK cell (CD49b+) negative selection enrichment kit from StemCell technologies according to the manufacture’s protocol. Purified NK cells (1x10^5^) were cultured in the presence or absence of 3x10^5^ bone marrow-derived macrophages (BMDM) or dendritic cells (BMDC) derived from uninfected wild type C57BL/6 mice or IFNγ^-/-^ mice with or without 1 ng/mL IFNγ, heat killed (hk) *C*. *rodentium* (3x10^6^ bacteria) and 8 ng/ml recombinant IL-2 for 24 h at 37°C in 5% CO_2_.

### Statistical analysis

Data was analyzed using GraphPad Prism (ver. 5.0d). Significance was assessed using the Student’s t test or ANAOVA as indicated in the figure legends. P-values less than 0.05 were considered significant.

## Supporting Information

S1 Fig *In vivo* depletion of CXCL9 results in loss of T cell homing. **(A)** C57BL/6 mice were infected with *C*. *rodentium* and on day 8 post infection CXCL9 levels in fresh feces, perfusion of the rectum, scraped mucus layer, and remaining colonic tissue were assessed by ELISA. All data is pooled from two separate experiments, n = 6. **(B)** Uninfected Rag1^-/-^ mice were injected with control rabbit IgG or anti-CXCL9 antibody. Fecal pellets were collected two days after the second injection and total rabbit IgG was determined by ELISA. **(C)** C57BL/6 mice were infected with *C*. *rodentium* and given either control rabbit IgG or anti-CXCL9 antibody. The number of CD3+ cells in the distal colon was quantified by immunohistochemical staining using an Image J script. **(D)** CD3+ immunohistochemistry images (200x) are representative of 2 experiments.
(TIF)

S2 Fig No significant changes to cytokine levels upon CXCL9-depletion *in vivo*. Six to eight week old Rag1^-/-^ mice were infected with *C*. *rodentium* for 10 days and given either anti-CXCL9 antibody or control rabbit IgG. Supernatants from colonic explants were measured by ELISA for the cytokines, **(A)** TNF-α, **(B)** IFN-γ, **(C)** IL12p40, and **(D)** IL10. All data is pooled from two separate experiments, n = 6 per group. Statistical significance was assessed utilizing the t-test.
(TIF)

S3 Fig Cecal histology in CXCL9-depleted animals. Rag1^-/-^ mice were infected with *C*. *rodentium* and administered either anti-CXCL9 antibody or control rabbit IgG. **(A)** Representative H&E-stained sections taken from the cecum (200x). Pathology scores in the cecum are quantified in **(B)**. Images and data are pooled from two experiments, n = 6 per group. **(C)** Localization of *C*. *rodentium* by immunohistochemical staining. Images (200X) are representative of 2 experiments, n = 6 per group. **(D)** Quantification of crypt invasion from immunohistochemical staining. Data is the means with standard errors from two separate experiments. Statistical significance was assessed utilizing the t-test.
(TIF)

S4 Fig Cecal histology in CXCR3^-/-^ animals containing CXCL9, or depleted of CXCL9. CXCR3^-/-^ mice were infected with *C*. *rodentium* and administered either anti-CXCL9 antibody or control rabbit IgG. **(A)** Representative H&E-stained sections taken from the cecum (200x) 10 days post-infection. Pathology scores in the cecum are quantified in **(B)**. Images and data are pooled from two experiments, n = 4 per group. **(C)** Localization of *C*. *rodentium* by immunohistochemical staining. Images (200X) are representative of 2 experiments, n = 4 per group. **(D)** Quantification of crypt invasion from immunohistochemical staining. Data is the means with standard errors from two separate experiments. Statistical significance was assessed utilizing the t-test.
(TIF)
