# Supplementary figures and images for: CXCL9 Contributes to Antimicrobial Protection of the Gut during Citrobacter rodentium Infection Independent of Chemokine-Receptor Signaling
Source: PLoS Pathog. 2015 Feb 2;11(2):e1004648. doi: 10.1371/journal.ppat.1004648 (PMC4333760; doi:10.1371/journal.ppat.1004648)

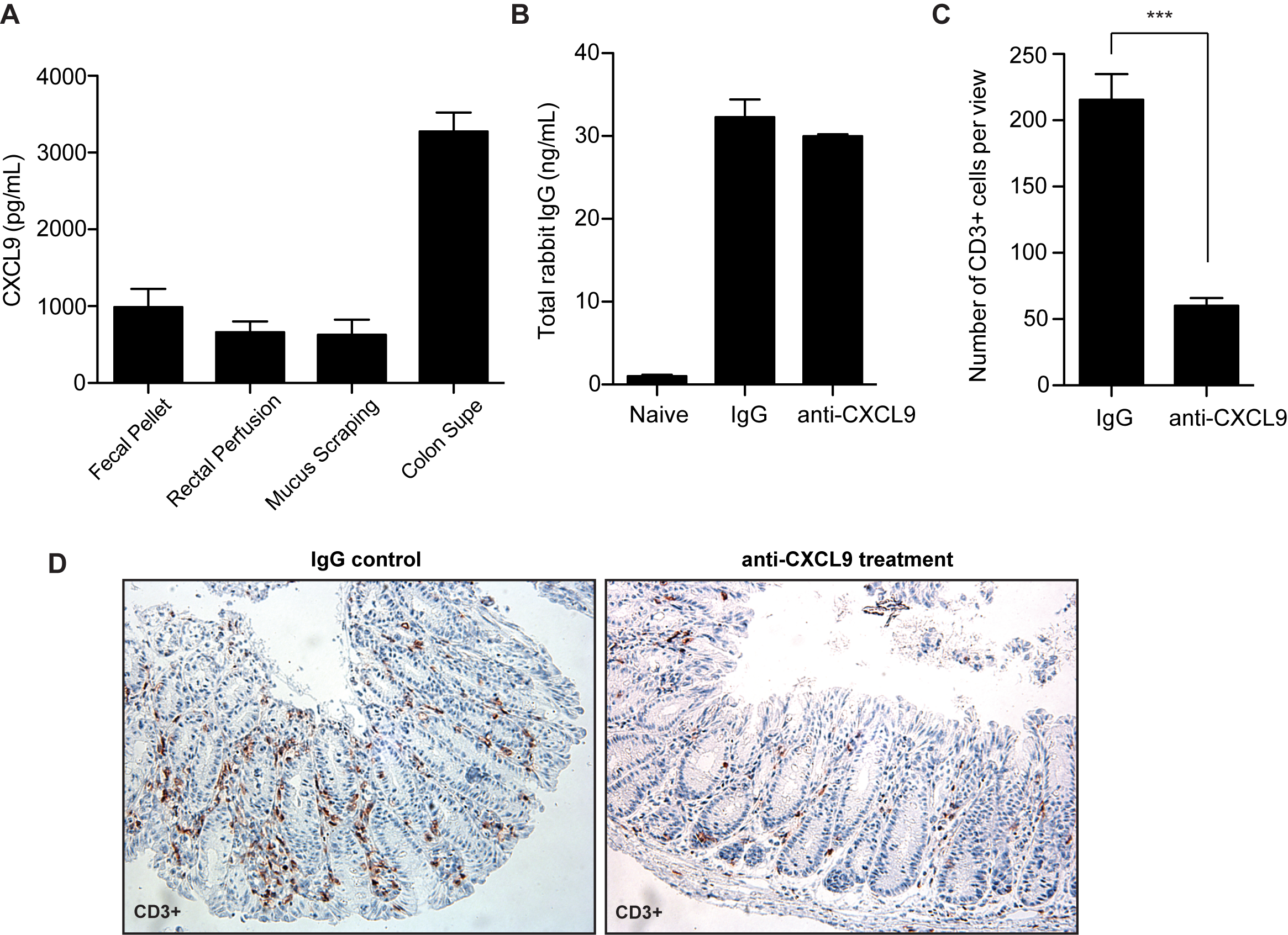

Supplement: S1 Fig — (A) C57BL/6 mice were infected with C. rodentium and on day 8 post infection CXCL9 levels in fresh feces, perfusion of the rectum, scraped mucus layer, and remaining colonic tissue were assessed by ELISA. All data is pooled from two separate experiments, n = 6. (B) Uninfected Rag1-/- mice were injected with control rabbit IgG or anti-CXCL9 antibody. Fecal pellets were collected two days after the second injection and total rabbit IgG was determined by ELISA. (C) C57BL/6 mice were infected with C. rodentium and given either control rabbit IgG or anti-CXCL9 antibody. The number of CD3+ cells in the distal colon was quantified by immunohistochemical staining using an Image J script. (D) CD3+ immunohistochemistry images (200x) are representative of 2 experiments. (TIF) [file ppat.1004648.s001.tif]

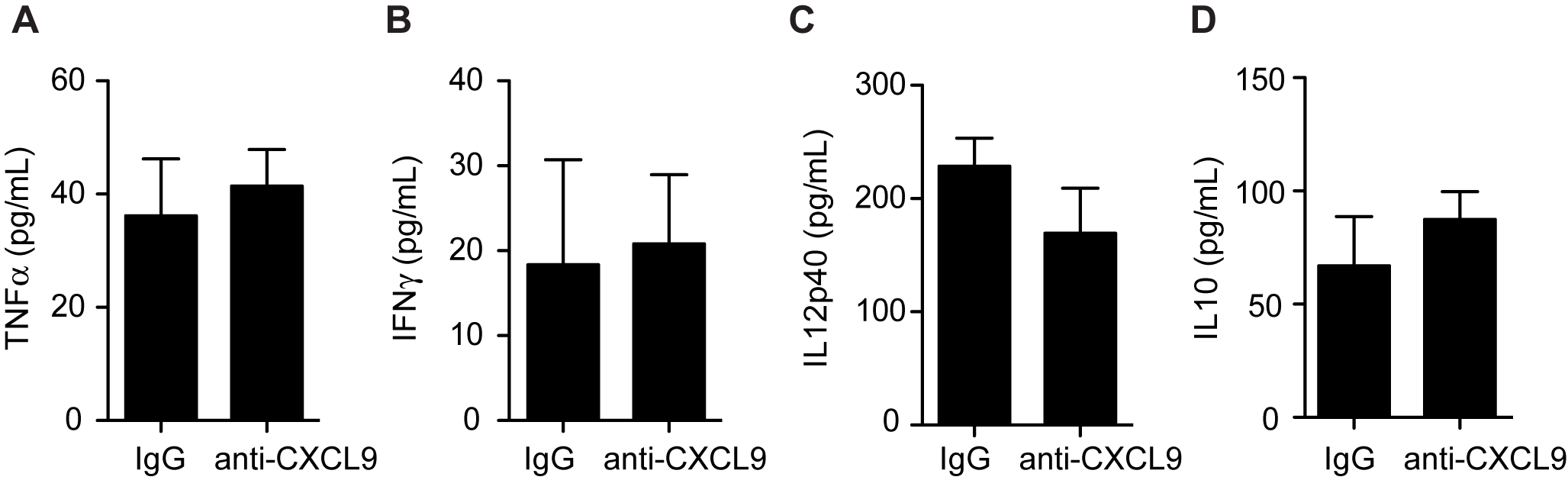

Supplement: S2 Fig — Six to eight week old Rag1-/- mice were infected with C. rodentium for 10 days and given either anti-CXCL9 antibody or control rabbit IgG. Supernatants from colonic explants were measured by ELISA for the cytokines, (A) TNF-α, (B) IFN-γ, (C) IL12p40, and (D) IL10. All data is pooled from two separate experiments, n = 6 per group. Statistical significance was assessed utilizing the t-test. (TIF) [file ppat.1004648.s002.tif]

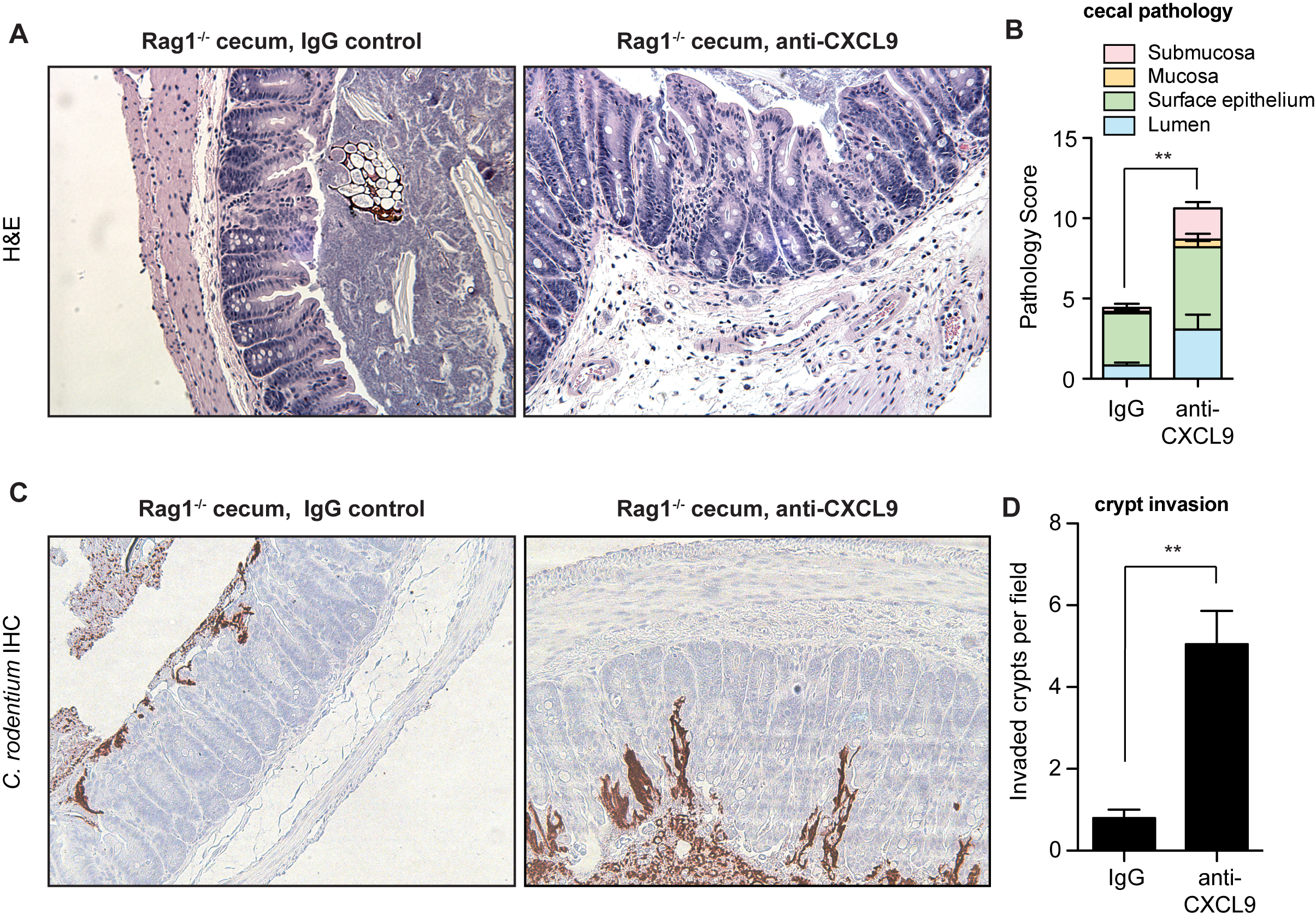

Supplement: S3 Fig — Rag1-/- mice were infected with C. rodentium and administered either anti-CXCL9 antibody or control rabbit IgG. (A) Representative H&E-stained sections taken from the cecum (200x). Pathology scores in the cecum are quantified in (B). Images and data are pooled from two experiments, n = 6 per group. (C) Localization of C. rodentium by immunohistochemical staining. Images (200X) are representative of 2 experiments, n = 6 per group. (D) Quantification of crypt invasion from immunohistochemical staining. Data is the means with standard errors from two separate experiments. Statistical significance was assessed utilizing the t-test. (TIF) [file ppat.1004648.s003.tif]

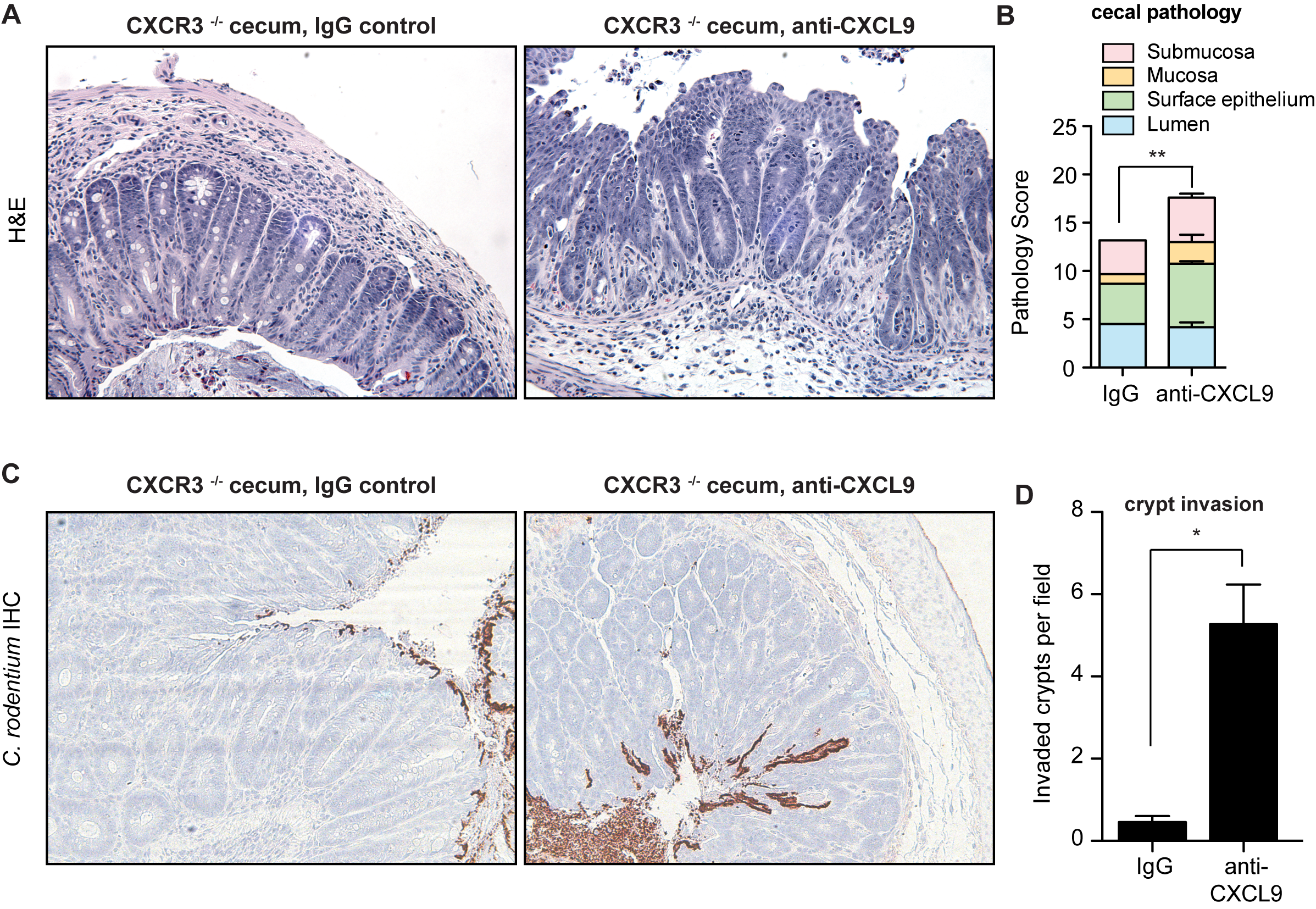

Supplement: S4 Fig — CXCR3-/- mice were infected with C. rodentium and administered either anti-CXCL9 antibody or control rabbit IgG. (A) Representative H&E-stained sections taken from the cecum (200x) 10 days post-infection. Pathology scores in the cecum are quantified in (B). Images and data are pooled from two experiments, n = 4 per group. (C) Localization of C. rodentium by immunohistochemical staining. Images (200X) are representative of 2 experiments, n = 4 per group. (D) Quantification of crypt invasion from immunohistochemical staining. Data is the means with standard errors from two separate experiments. Statistical significance was assessed utilizing the t-test. (TIF) [file ppat.1004648.s004.tif]
